# Functional characterization of four opsins and two G alpha subtypes co-expressed in the molluscan rhabdomeric photoreceptor

**DOI:** 10.1186/s12915-023-01789-7

**Published:** 2023-12-18

**Authors:** Ryota Matsuo, Mitsumasa Koyanagi, Tomohiro Sugihara, Taishi Shirata, Takashi Nagata, Keiichi Inoue, Yuko Matsuo, Akihisa Terakita

**Affiliations:** 1 International College of Arts and Sciences, Fukuoka Women’s University, 1-1-1 Kasumigaoka, Higashi-Ku, Fukuoka, 813-8529 Japan; 2 Department of Biology, Graduate School of Science, Osaka Metropolitan University, 3-3-138 Sugimoto, Sumiyoshi-Ku, Osaka, 558-8585 Japan; 3 Department of Biology and Geosciences, Graduate School of Science, Osaka City University, 3-3-138 Sugimoto, Sumiyoshi-Ku, Osaka, 558-8585 Japan; 4 The OMU Advanced Research Institute of Natural Science and Technology, Osaka Metropolitan University, 3-3-138 Sugimoto, Sumiyoshi-Ku, Osaka, 558-8585 Japan; 5 The Institute for Solid State Physics, The University of Tokyo, Kashiwa, Japan; 6 PRESTO, Japan Science and Technology Agency, Kawaguchi, Japan

**Keywords:** co-expression, *Lehmannia*, Opsin, Light adaptation, Spectral sensitivity, Phototranduction, G protein signaling

## Abstract

**Background:**

Rhabdomeric photoreceptors of eyes in the terrestrial slug *Limax* are the typical invertebrate-type but unique in that three visual opsins (Gq-coupled rhodopsin, xenopsin, Opn5A) and one retinochrome, all belonging to different groups, are co-expressed. However, molecular properties including spectral sensitivity and G protein selectivity of any of them are not determined, which prevents us from understanding an advantage of multiplicity of opsin properties in a single rhabdomeric photoreceptor. To gain insight into the functional role of the co-expression of multiple opsin species in a photoreceptor, we investigated the molecular properties of the visual opsins in the present study.

**Results:**

First, we found that the fourth member of visual opsins, Opn5B, is also co-expressed in the rhabdomere of the photoreceptor together with previously identified three opsins. The photoreceptors were also demonstrated to express Gq and Go alpha subunits. We then determined the spectral sensitivity of the four visual opsins using biochemical and spectroscopic methods. Gq-coupled rhodopsin and xenopsin exhibit maximum sensitivity at ~ 456 and 475 nm, respectively, and Opn5A and Opn5B exhibit maximum sensitivity at ~ 500 and 470 nm, respectively, with significant UV sensitivity. Notably, in vitro experiments revealed that Go alpha was activated by all four visual opsins, in contrast to the specific activation of Gq alpha by Gq-coupled rhodopsin, suggesting that the eye photoreceptor of *Limax* uses complex G protein signaling pathways.

**Conclusions:**

The eye photoreceptor in *Limax* expresses as many as four different visual opsin species belonging to three distinct classes. The combination of opsins with different spectral sensitivities and G protein selectivities may underlie physiological properties of the ocular photoreception, such as a shift in spectral sensitivity between dark- and light-adapted states. This may be allowed by adjustment of the relative contribution of the four opsins without neural networks, enabling a simple strategy for fine-tuning of vision.

**Supplementary Information:**

The online version contains supplementary material available at 10.1186/s12915-023-01789-7.

## Background

Photopigment serves as a photon capturing molecule in the photoreceptors of animals and consists of a G protein-coupled receptor opsin and a chromophore retinal. Thousands of opsins have been identified from various animals and are phylogenetically and functionally classified into several groups, many of which are characterized by the G protein coupling selectivity, such as Gt-, Gq-, Go-, Gs-coupled opsins, and so on [1]. Usually, only a single species of opsin is expressed in a photoreceptor, and it dictates the spectral sensitivity of the photoreceptor. However, there are several instances where multiple opsin species are co-expressed in the photoreceptors of arthropods [2–5]. The co-expressed opsins are all categorized into Gq-coupled rhabdomeric opsins, and have different spectral sensitivities. Therefore, the co-expression of opsins has been thought to contribute to the broadening of spectral sensitivity of the photoreceptors.

The terrestrial slug *Limax* possesses a highly sensitive camera-type eye on the tip of the superior tentacle (ST). We recently reported the co-expression of four opsins in the eye photoreceptor of *Limax* [6]. In contrast to the reports on the co-expression of two or more opsins in some arthropods as mentioned above, the slug’s photoreceptor was intriguing in that the co-expressed opsins belonged to the distinct opsin groups. Based on the molecular phylogenetic classification, the four opsins were named *Limax* Gq-coupled rhodopsin (Gq-rhodopsin), xenopsin, Opsin5A (Opn5A), and retinochrome. We also identified Opsin5B (Opn5B) whose expression profile has not been analyzed yet. Gq-rhodopsin has been considered the major opsin functioning in invertebrate eyes [7–9]. Xenopsin is a recently identified opsin in mollusks, brachiopods, and platyhelminthes [10–14], and it is reported to be a visible light-sensitive opsin that regulates cAMP signaling [12, 15]. Opn5A and Opn5B are similar to vertebrate Opn5 at the amino acid sequence level, although Opn5-like opsins of invertebrates have been less investigated [16]. Retinochrome is not a signaling-competent opsin but a retinal photoisomerase, present in mollusks [17–19].

Three of the four signaling-competent opsins (Gq-rhodopsin, xenopsin, and Opn5A) were shown to be co-expressed in the rhabdomere of Type-I photoreceptors that are the principal photoreceptor in the retina equipped with highly developed microvilli [6, 20–23]. Retinochrome was present in the cell body [6, 18]. To elucidate the functional role of the co-expression of opsins belonging to different classes, the spectral sensitivities of each opsin species should be determined first. Moreover, the locus of expression of an unexamined opsin species, Opn5B, also needs to be determined.

In the present study, we generated a specific antibody against the C-terminus of Opn5B of *Limax* and demonstrated that Opn5B is the fourth member of visual opsins co-expressed in the rhabdomere of Type-I photoreceptors in the retina. Then the spectral sensitivities were determined for all four visual opsins. We also uncovered G proteins expressed in the photoreceptors and analyzed the coupling competencies of opsins to the G proteins in a pairwise manner in vitro. We discuss the role of the co-expression of multiple opsins in the context of the shift in the spectral sensitivity of the *Limax* eye depending on light- and dark adapted states of the animal.

## Results

### Opn5B protein is colocalized with Gq-rhodopsin in the eye

First, we generated a polyclonal antibody against the C-terminus of Opn5B. The purified antibody specifically labeled HEK293 cells transfected with an expression vector harboring the open reading frame (ORF) of Opn5B that was tagged with 6 × His in the N-terminus (Additional file 1: Fig. S1), supporting the validity of the generated anti-Opn5B antibody.

**Fig. 1:**
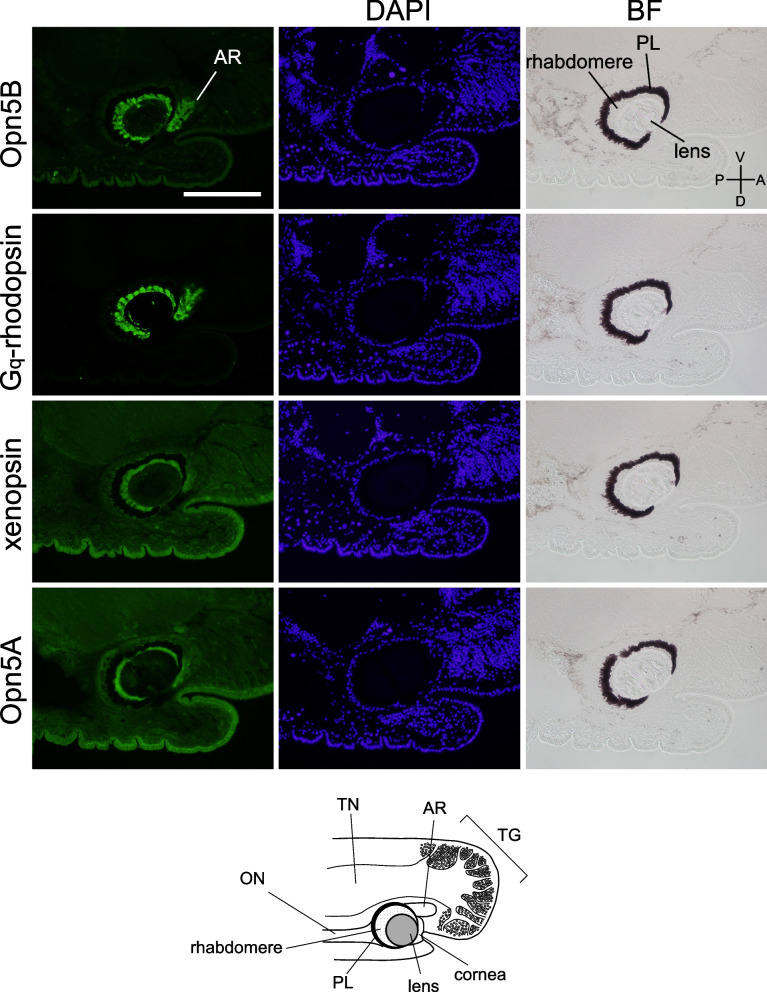
Immunohistochemical staining of opsin proteins. Serially sectioned superior tentacles (10 μm thick) were stained with specific antibodies. Below is a cartoon explaining the anatomy of the superior tentacle. Scale bar: 200 μm. A, anterior; P, posterior; D, dorsal; V, ventral. AR, accessory retina; PL, pigment layer; TG, tentacular ganglion; TN, tentacular nerve; ON, optic nerve; BF, bright-field image

Next, we investigated the localization of the opsin proteins in the eye of *Limax*. As previously demonstrated [6], immunohistochemical staining of Gq-rhodopsin, xenopsin, and Opn5A in the serial section (10 μm thick) of the eye revealed the immune signals in the rhabdomeric region (Fig. 1). In addition, we demonstrated the immunoreactivity of Opn5B in the same region (Fig. 1). The immunoreactive signals were also evident in the accessory retina (AR), which lacks the pigment layer [24, 25].

**Fig. 2:**
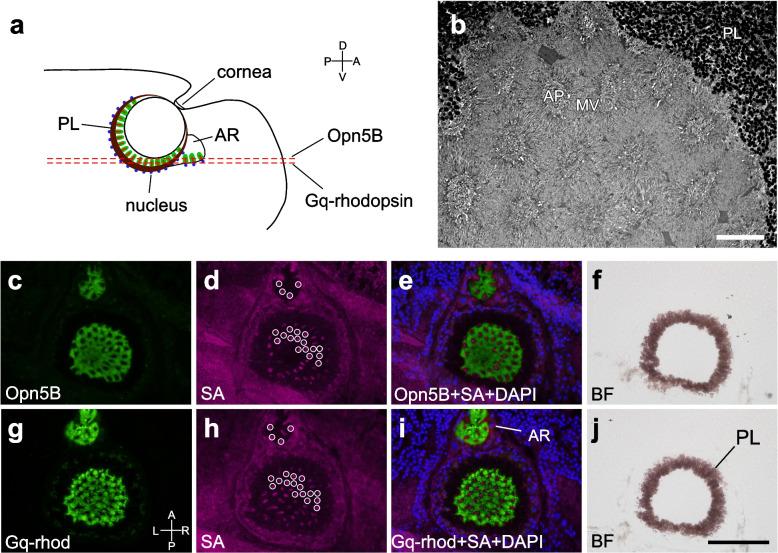
Co-expression of Opn5B and Gq-rhodopsin in eye photoreceptors. **a** A schema explaining the cutting planes of the superior tentacle viewed from the right side. **b** An electron microscope image of the section of the retina that was cut at a similar position to the broken lines in (a), showing cross-sectioned rhabdomeres of the Type-I photoreceptors. Lower left panels (**c**, **g**) are immunofluorescence of Opn5B or Gq-rhodopsin. Next right panels (**d**, **h**) are fluorescence of streptavidin, which stains the apical protrusions of the Type-I eye photoreceptor. Some streptavidin signals are circumscribed with white circles to show similar distribution patterns between (d) and (h). (**e**) and (**i**) are the superimposed images of the left two panels on DAPI nuclear images. (**f**) and (**j**) are the bright-field images of the left panels. (c-f) and (g-j) are the pair of 6 μm-thick serial sections. Scale bar: (b) 10 μm, (j) 100 μm. A, anterior; P, posterior; D, dorsal; V, ventral; R, right; L, left. AR, accessory retina; AP, apical protrusion; MV, microvilli; PL, pigment layer; SA, streptavidin; BF, bright-field image

We also investigated whether the Opn5B protein is co-expressed with other opsin proteins. Because these anti-opsin antibodies were all raised in rabbits, it was difficult to dually immunostain different opsins in the same sections. Therefore, we exploited the large dimension of the Type-I photoreceptors along the apical protrusion and prepared thin sections (6 μm thick) of the eye that were serially cut in a horizontal direction (Fig. 2a, b). An apical protrusion of the Type-I photoreceptor appeared in the two neighboring sections because of their small thickness (Fig. 2a). This was revealed by the similar distribution patterns of the cross-sectioned apical protrusions that were known to be stained by streptavidin (Fig. 2d, h, [6]). As shown in Fig. 2e and i, both immune signals of Opn5B and Gq-rhodopsin circumscribe the common apical protrusions, suggesting that both proteins are localized in the microvilli of the same Type-I photoreceptors. Therefore, four kinds of visual pigments (Gq-rhodopsin, xenopsin, Opn5A, and Opn5B) are all localized to the rhabdomere of Type-I photoreceptors.

### Expression of Gq and Go alpha subunits in the retina

**Fig. 3:**
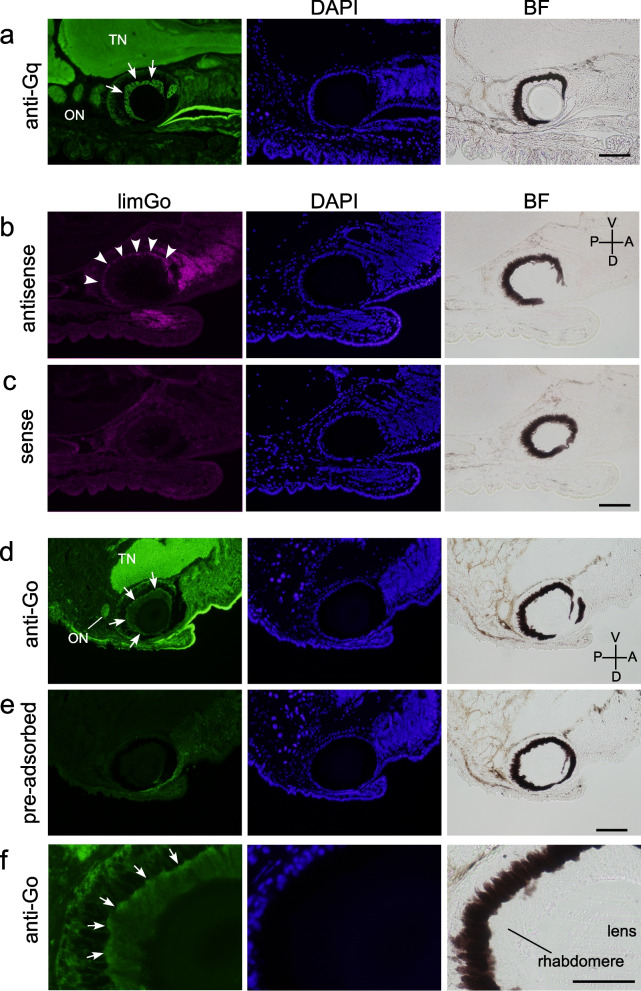
Expression of G protein alpha subunits in the retina. **a** Immunostaining of limGα_q_. White arrows indicate the signals in the rhabdomeric region of the eye. **b** Fluorescence in situ hybridization with the antisense probe for limGα_o_ mRNA. Arrow heads indicate the positive signals in the cell body layer. **c** Hybridization with the sense probe did not exhibit signals. **d** Immunostaining of limGα_o_ showing the region near the eye. White arrows indicate the signals in the rhabdomeric region of the eye. **e** Pre-adsorption with MBP-limGα_o_ protein diminished the immunoreactive signals. **f** A magnified image of (d) focusing on the rhabdomeric region (white arrows). Scale bars: 100 μm. TN, tentacular nerve; ON, optic nerve; BF, bright-field image. A, anterior; P, posterior; D, dorsal; V, ventral

Then, we analyzed the expression of alpha subunits of trimeric G proteins to obtain candidates for G protein signaling driven by the multiple kinds of opsins in the photoreceptor cell. It is well known that Gq-mediated signaling plays a pivotal role in invertebrate visual systems [7–9, 26]. Therefore, we first focused on the alpha subunit of Gq. Immunohistochemical staining with commercially obtained anti-human Gα_11_ antibody, of which specific reactivity to the alpha subunit of *Limax* Gq (limGα_q_) was confirmed (Additional file 2: Fig. S2a-c), showed the presence of limGα_q_ in the rhabdomere of Type-I photoreceptors (Fig. 3a), which is consistent with the expression of Gq-rhodopsin in the photoreceptor cell.

Recently, xenopsins were suggested to activate Gi when transfected into cultured mammalian cells [12, 15]. Avian and mammalian Opn5 were also shown to activate Gi in vitro [27–29]. Co-expression of xenopsin, Opn5A, Opn5B, and Gq-coupled rhodopsin in a single photoreceptor implies the presence of G protein signaling other than Gq. However, the expression of the alpha subunit of Gi was not detected by in situ hybridization (Additional file 3: Fig. S3). In contrast, the expression of the alpha subunit of Go type G protein (limGα_o_), a type of the Gi subgroup, was detected by in situ hybridization (Fig. 3b, c). The protein of limGα_o_ was also immunohistochemically detected with commercially obtained anti-bovine Gα_o_ that was confirmed to react to limGα_o_ using HEK293 cells transfected with pEGFP-limGα_o_ (Additional file 2: Fig. S2d-g), although the immunoreactivity was detected in both the rhabdomere and cell body layer of the retina (Fig. 3d, f). The validity of the immunoreactivity was further confirmed by pre-adsorption of the antibody with limGα_o_ fused to the C-terminus of maltose binding protein (MBP) (Fig. 3d, e).

### Spectral sensitivity of opsins

**Fig. 4:**
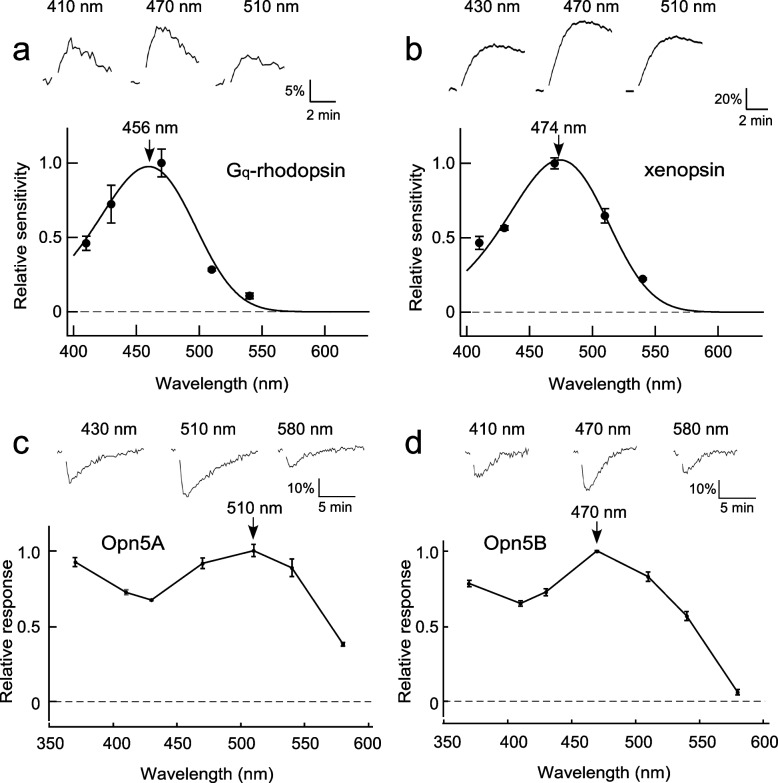
Spectral sensitivities of opsin-based pigments determined by GloSensor cAMP-dependent luciferase reporter assay. **a**, **b** The sensitivities of Gq-rhodopsin and xenopsin were determined by HAS, based on the light-induced increase in the intracellular cAMP concentration ([cAMP]_i_), using chimeric opsin containing the intracellular domain of Gs-coupled jellyfish opsin. The sensitivity curve of each opsin was estimated by fitting the sensitivity values with a template of the rhodopsin spectrum (i.e., rhodopsin nomogram). **c**, **d** The relative amplitudes of the Opn5A and Opn5B responses were determined by the decrease in the [cAMP]_i_ in the cultured cells expressing wild-type Opn5A or Opn5B. Upper traces are examples of light-dependent changes in the relative [cAMP]_i_ levels. Error bars: ± SE (*n* = 3)

It is necessary to elucidate the spectral sensitivities of opsins expressed in the same photoreceptor cells to understand their contributions to the photoreceptor functions. We first investigated spectral sensitivities of opsins by heterologous action spectroscopy (HAS), which is based on light-induced increases of intracellular cAMP concentrations ([cAMP]_i_) in cultured cells expressing opsins and can provide detergent-free absorption spectra [30, 31]. Since the HAS method requires Gs-activation ability for opsins, chimeric mutants with the third cytoplasmic loop of the jellyfish Gs-coupled opsin were generated for each opsin [32]. In the case of Gq-coupled rhodopsin and xenopsin, cultured cells expressing the Gs-coupled chimeras exhibited light-induced cAMP increases. We then obtained the relative sensitivities of Gq-coupled rhodopsin and xenopsin at each wavelength based on the response amplitudes (Fig. 4a, b) and the dose–response (light intensity-response) curves (Additional file 4: Fig. S4). The relative response values were fitted with a rhodopsin nomogram having absorption maxima at 456 and 474 nm, respectively (Fig. 4a, b).

In contrast, the cultured cells expressing Gs-coupled chimeras for Opn5A or Opn5B did not exhibit light-induced clear increases of cAMP. However, the Opn5A or Opn5B “wild-type”-expressing cultured cells showed light-induced cAMP decreases (upper traces in Fig. 4c, d), as are vertebrate Opn5 [27, 29], which enables their relative response curves to be obtained [33]. As vertebrate Opn5s have been demonstrated to be UV-responsive [27–29], the responses to a shorter wavelength of light (370 nm) were also measured in the case of *Limax* Opn5s. Thus, we obtained the relative light responses to seven different wavelengths for Opn5A or Opn5B-expressing cells based on the light-induced decreases of cAMP. Surprisingly, Opn5A and Opn5B exhibited maximum responses at approximately 510 and 470 nm, respectively, in addition to responses in the UV region (Fig. 4c, d). Each Glosensor assay was independently performed three times, and we could obtain reproducible data (Fig. 4, Additional file 5: Table S1).

Mock-transfected HEK293S cells did not show any UV responses (Additional file 6: Fig. S5), indicating that the UV responses are not artifacts reflecting the HEK293S cells themselves or phosphorescence of plastic dishes for the cultured cells. One explanation for the comparable response amplitudes between the UV and visible region is that the UV and visible responses could be based on the absorption derived from two states of retinal chromophore in the opsin molecules, deprotonated and protonated states of the retinylidene Schiff base, which are basic spectral tuning mechanisms for UV- and visible light-sensitivities of opsin-based pigments, respectively (See Discussion section).

**Fig. 5:**
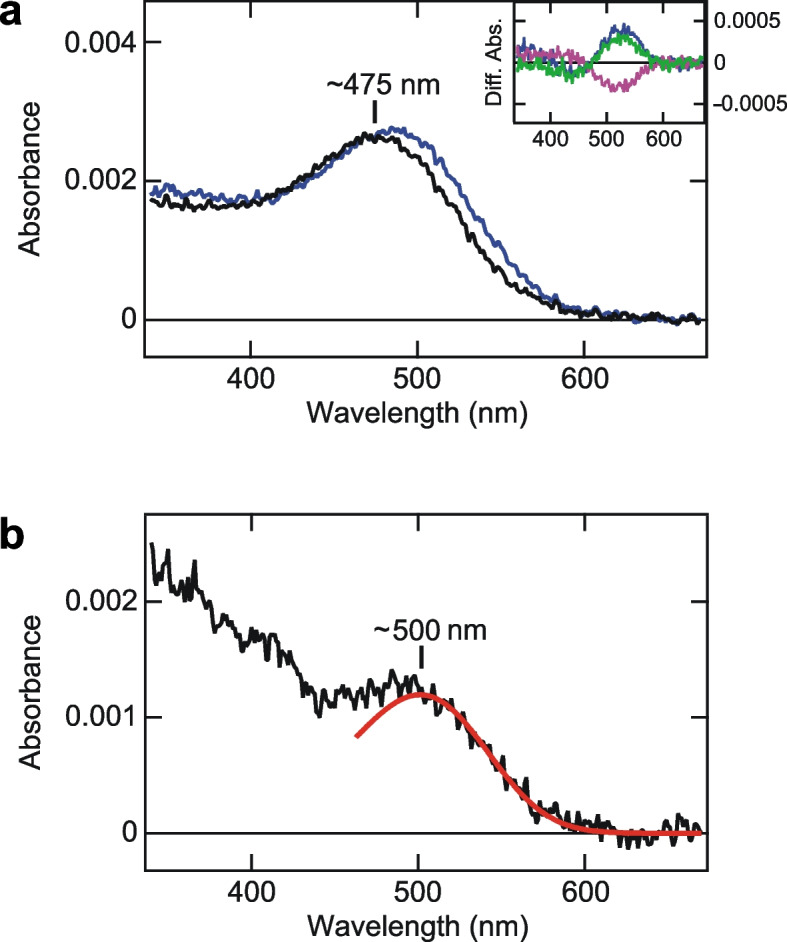
Absorption spectra of *Limax* xenopsin and Opn5A. **a** The absorption spectrum of xenospin in the dark (black curve) and after blue light irradiation (blue curve). (inset) The difference absorption spectra generated by subtracting absorbance at each wavelength before from after blue light irradiation (blue curve), before from after subsequent orange light irradiation (magenta curve), and before from after subsequent second blue light irradiation (green curve). The green curve was the mirror image of the magenta curve, indicating the bistability of xenopsin. **b** The absorption spectrum of Opn5A (black curve) fitted with the rhodopsin nomogram that has an absorption maximum of ~ 500 nm (red curve). Note that absorbance under 430 nm may be affected by scattering from impurities

We also successfully purified recombinant opsin-based pigments for xenopsin and Opn5A. Spectroscopic analysis revealed that xenopsin forms a photopigment with an absorption maximum at ~ 475 nm (Fig. 5a), which shows a good agreement with the spectral sensitivity estimated by HAS (Fig. 4b), even in the presence of detergent. Blue light irradiation of xenopsin resulted in an increase and decrease of the absorbance at ~ 530 and ~ 450 nm, respectively (Fig. 5a, a blue curve in inset), indicating the photoconversion of xenopsin to the photoproduct with a red-shifted absorption spectrum. Subsequent orange light irradiation caused the opposite reaction, which indicates the photoconversion of a part of the photoproduct to the dark state (Fig. 5a, a magenta curve in inset). The photoconversion repeatedly occurred by blue and orange light irradiation, showing that the *Limax* xenopsin is a bistable photoconvertible opsin with two stable states, like many other opsins, including xenopsins of other animals [1, 15]. Spectroscopic analysis of Opn5A revealed that *Limax* Opn5A has absorbance in the visible light region (Fig. 5b). Since the absorption spectrum was affected by absorbance derived from impurities because of the low expression level of Opn5A, the rhodopsin nomogram [34] was fitted to estimate the absorption spectrum of Opn5A in the visible light region. The nomogram fitting revealed that the wavelength for the visible absorption maximum of Opn5A is ~ 500 nm, which supports the spectral sensitivity based on light-induced decreases of cAMP in Opn5A-expressing cultured cells (Fig. 4c). However, it was hard to discuss about the absorbance in the UV region because of the scattering due to impurities.

Although retinochrome is known to function as photoisomerase rather than a signaling-competent opsin in cephalopods [17, 35], we here re-evaluated whether it holds true for *Limax* retinochrome. A full length retinochrome was expressed in HEK293S cells, and the competence of its light-dependent signal activation was examined. However, green light irradiation did not induce either the change of [Ca^2+^]_i_ or [cAMP]_i_, suggesting that retinochrome of *Limax* does not drive Gi/o or Gq signaling pathway (Additional file 7: Fig. S6).

These results revealed that four kinds of visual opsins co-expressed in the rhabdomere of Type-I photoreceptor cells have spectral sensitivities ranging from blue (456 nm) to green (500 nm), and two Opn5s have a high sensitivity to UV light.

### Interaction of opsins with G proteins

**Fig. 6:**
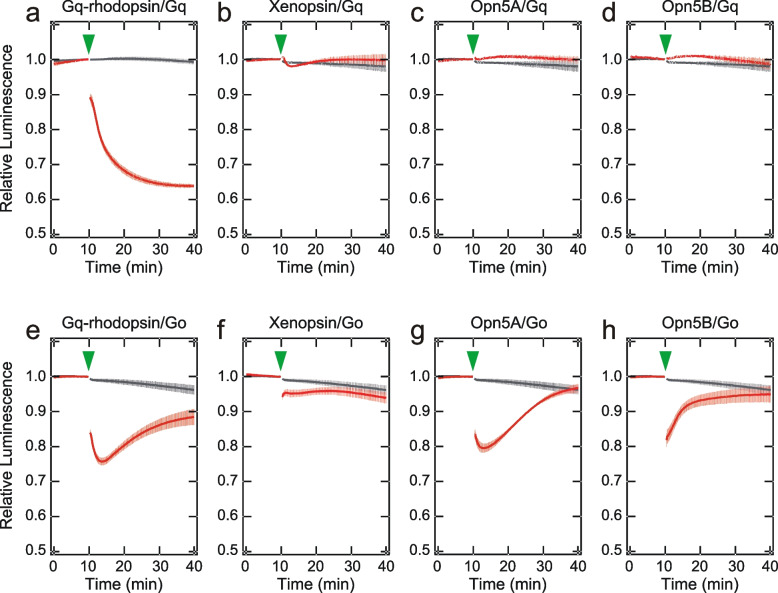
Gq and Go activation abilities of *Limax* opsins measured by NanoBiT-G-protein dissociation assay. (**a-d**) Gq activation assay for Gq-rhodopsin (a), xenopsin (b), Opn5A (c), and Opn5B (d). (**e–h**) Go activation assay for Gq-rhodopsin (e), xenopsin (f), Opn5A (g), and Opn5B (h). Changes in the luminescence of cultured cells expressing each opsin (red) and no opsin (black) were shown. Error bars indicate SE (*n* = 3). Arrow heads indicate the light stimuli

Given that at least two different G proteins, Gq and Go, are present in the rhabdomeres, it is intriguing to know in which combination the opsin-G protein interaction occurs. We examined Gq and Go activation abilities of *Limax* opsins using a NanoBiT-G-protein dissociation assay [36]. In the case of the Gq activation assay, a light-induced decrease of luminescence, which indicates a dissociation of Gα_q_ from Gβγ due to heterotrimeric G protein activation, was observed as expected in cultured cells expressing Gq-rhodopsin, whereas it was not observed in those expressing other opsins (Fig. 6a-d, n = 3). Surprisingly, in the case of the Go activation assay, cultured cells expressing any of the four opsins, including Gq-rhodopsin, reproducibly exhibited a significant light-induced dissociation of Gα_o_ from Gβγ, showing their Go activation ability (Fig. 6e-h, n = 3). Individual data in the NanoBit-G protein dissociation assay are displayed in Additional file 8 (Fig. S7). In contrast, jellyfish opsin, which is known to couple to Gs but not to Go signaling pathway [32], did not reduce luminescence in NanoBiT-G-protein dissociation assay (Additional file 9: Fig. S8), excluding the possibility that Go is promiscuously activated by any opsin in this system. These results suggest that, in addition to the Gq-mediated phototransduction cascade, the Go-mediated phototransduction cascade is cooperatively triggered by four opsins in *Limax*.

## Discussion

In the retina of the molluscan eye, Gq-rhodopsin has been considered the principal opsin responsible for vision [1]. However, we demonstrated the co-expression of as many as four different opsin species that belong to different groups, Gq-rhodopsin, xenopsin, Opn5A, and Opn5B, in the photoreceptor of the eye of the slug *Limax*. They were all expressed in the rhabdomere of the Type-I photoreceptor, which is morphologically distinguished from other retinal cells with highly developed microvilli [20–22, 37].

Co-expression of opsins in the same class has been reported relatively frequently in invertebrate eyes [3–5, 38, 39]. Co-expression of opsins belonging to different classes has also been reported in non-visual photoreceptors of vertebrates. For example, VA/VAL opsin and TMT-opsin are co-expressed in the central interneurons of medaka and zebrafish conferring photosensitivity on these neurons [40]. Pinopsin or parapinopsin are colocalized with parietopsin in the outer segments of the photoreceptors in the parietal eye of lizard and iguana, contributing to generation of color opponency [41, 42]. Colocalization of parapinopsin and parietopsin has also been found in zebrafish pineal organ [43]. Some recent studies have reported the co-expression of opsins in different classes in the eye photoreceptor of some invertebrates, such as Go-opsin and Gq-rhodopsin in the eye of *Platynereis* [44], Gq-rhodopsin and Gi/Go-coupled Opn3 in mosquito eye [45], and xenopsin and Gq-rhodopsin in the larval eye of *Leptochiton* [11]. But as far as we know, the finding of co-expression of as many as four different visual opsins is unprecedented.

For the *Limax* Gq-rhodopsin, the peak sensitivity (λ_max_) was 456 nm (Fig. 4a). This wavelength is slightly shorter compared to the previously reported λ_max_ of Gq-rhodopsin extracts from cephalopod retinas, i.e., 480 nm in *Octopus vulgaris*, 482 nm in *Todarodes pacificus*, 484 nm in the firefly squid *Watasenia scintillans*, 499 nm in the European common squid *Alloteuthis subulata*, and 494 nm in the long-finned squid *Loligo forbesi* [46, 47]. Even in gastropods, the λ_max_ of the Gq-rhodopsin-containing fraction was 474 nm in a strawberry conch *Conomurex luhuanus* [19]. The discrepancies between these values and our HAS measurements may be attributed to the difference in animal species, and the shortest λ_max_ of *Limax* Gq-rhodopsin may relate to their terrestrial lifestyle. Alternatively, these differences may be partly due to the presence of other opsins (Opn5A or xenopsin, etc.) in the extracts, which may have contributed to the shift of λ_max_ to a longer wavelength range in these previous biochemical studies.

We evaluated the absorption spectrum of *Limax* xenopsin and revealed that xenopsin is a blue-sensitive opsin (λ_max_ =  ~ 475 nm) (Figs. 4b, 5a). The result, together with the wavelengths for the maximum sensitivity of Gq-rhodopsin (456 nm, Fig. 4a) and the maximum response of Opn5B (~ 470 nm, Fig. 4d), indicates that three opsins respond to a similar color of light (blue) in the same photoreceptor cell. In contrast, our HAS measurement, as well as spectroscopic analysis of recombinant pigment, demonstrated that Opn5A of *Limax* has a sensitivity peak at ~ 500 nm, in a green light region (Figs. 4c, 5b).

The majority of vertebrate Opn5 are only sensitive to UV [27–29, 48], and some Opn5-like opsins in vertebrates, such as Opn5 in quail, Opn5L1 in chicken, and Opn6a in zebrafish, were reported to have peak sensitivities in the visible light range [48–50]. Therefore, the peak sensitivities of Opn5A and B in the blue to green region in addition to UV sensitivity in the cultured cells, namely double-peaks, are intriguing. Their double-peaks were observed in relative responses with cultured cells but not clearly in the absorption spectrum of the purified Opn5A because of scattering in the shorter wavelength region (less than 400 nm). Such a double-peak was also suggested in a previous report, where zebrafish Opn7d, which is also related to other vertebrate Opn5s at the amino acid sequence level, exhibits biphasic absorbance peaks in the UV and visible light ranges [48]. Accumulated evidence has established that a retinal chromophore is bound to the highly conserved Lys residue in known animal opsins via a retinylidene Schiff base. The Schiff base forms an equilibrium between deprotonated and protonated forms, which are basic spectral tuning mechanisms for UV- and visible light-sensitive opsin-based pigments, respectively (see [51]). In visible light-sensitive opsin-based pigment, the protonation of the Schiff base is stabilized by a negatively charged amino acid residue called a counterion near the Schiff base, in most cases Glu. In this point of view, one explanation is that the UV and visible sensitivity of Opn5A and B in cultured cells (Fig. 4) could be derived from a mixture of the deprotonated and protonated states of the retinylidene Schiff base, in which the two state might be regulated by amino acid residues near the chromophore including the counterion. This idea is supported by previous findings with some counterion-related mutant opsins. Similar double peaks were observed in cultured cells (Fig. 2 of [52]) and some detergent conditions [53] by eliminating or weakening the effects of the counterion or its complex on stabilization of the protonation. The detailed mechanism for the double-peak is a next interesting issue.

We detected the expression of Gα_q_ and Gα_o_ in photoreceptor cells (Fig. 3). It is well known that Gq-mediated signal transduction is the primary visual signaling in invertebrate rhabdomeric photoreceptor cells [7–9, 26], whereas Go-mediated signal transduction was found in a limited number of species [44, 54], although some ciliary photoreceptor cells employ Go-signaling cascades [41, 55]. Our NanoBiT assay confirmed that *Limax* Gq-rhodopsin activates the Gq-signaling cascade (Fig. 6a). Unexpectedly, the Gq-rhodopsin also activates the Go-signaling cascade, extending the Gq-rhodopsin function in signal transduction (Fig. 6e). We also demonstrated for the first time that xenopsin, Opn5A, and Opn5B activate the Go-signaling cascade (Fig. 6f, g, h). This result is consistent with the light-dependent reduction of [cAMP]_i_ in the GloSensor assay (Fig 4c, d) because opsins activating Go are known to also activate Gi [56, 57]. Our results are also consistent with previous reports on the light-dependent decrease in [cAMP]_i_ in the HEK293 cells expressing xenopsin or vertebrate Opn5 [12, 15, 27, 29].

We recently reported that the spectral sensitivity of the eye of *Limax valentianus* shifts between dark- and light-adapted states [6]. Similar phenomenon has also been reported be Suzuki et al. (1979) in *Limax flavus* [58]. Light adaptation-dependent shift in the sensitivity peak would be advantageous because it enables efficient detection of light depending on the change of the spectral compositions of ambient light during the time of day (dawn/dusk or midday). Especially, it is very important for the slugs, which are nocturnal, to detect ambient light efficiently under starlight. It would be noteworthy that the relative number of photons is more abundant in > 560 nm range in the spectrum of starlight compared to midday [59, 60]. The habitat of the slugs may also be relevant. On the ground in the forest, the spectral composition of ambient light is affected by the covering leaves and grasses, which have relatively large transmittance for longer wavelength range (> 500 nm) [61, 62]). Therefore, it would be more advantageous for slugs to adjust the spectral sensitivity of their eyes for the detection of longer wavelength light in dim environment.

The co-expression of several kinds of opsins with different spectral sensitivities and G protein coupling selectivities may provide one possible explanation that adjustment of the relative contribution of the four opsins could shift the spectral sensitivity. In the dark-adapted state, the sensitivity of the electroretinogram (ERG) response is broad, with a peak at 480 nm [6]. This is a slightly longer wavelength compared with the λ_max_ of xenopsin and Opn5B, and thus Opn5A (λ_max_ = 500 nm) may also contribute to the generation of ERG during a dark-adapted state. In contrast, the light-adapted state with a peak sensitivity of the ERG response at 420 nm may be explained by the higher contribution of Gq-rhodopsin (λ_max_ = 456 nm) and the UV sensitivity of Opn5s (Fig. 4c, d).

**Fig. 7:**
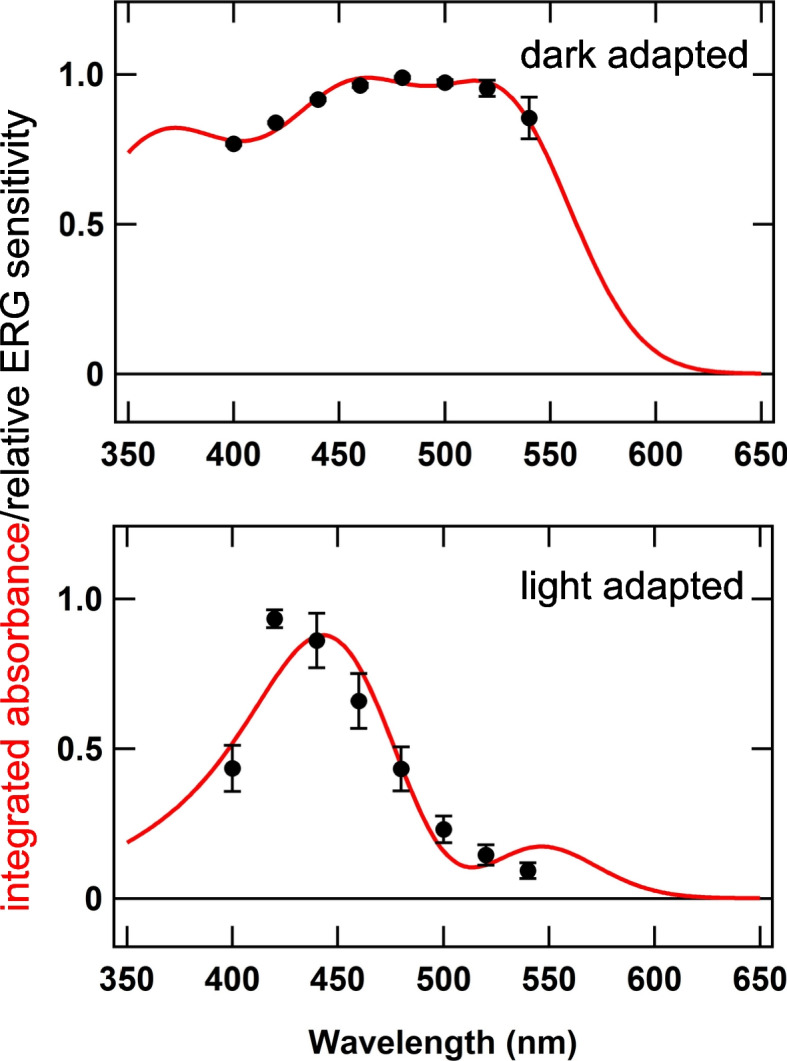
Plausible contribution of different opsins to ERG during dark and light adaptation. Black dots and error bars (± SE, *n* = 7 for dark-adapted, *n* = 8 for light-adapted) indicate the relative ERG sensitivities reproduced from Matsuo et al. (2019) [6]. Red lines are the linear summations of four rhodopsin nomograms (Additional file 10: Fig. S9), experimentally obtained for Gq-rhodopsin (Fig. 4a), xenopsin (Fig. 4b), Opn5A (Fig. 5b), estimated based on the wavelength of the maximal relative response of Opn5B (Fig. 4d) and using 360 nm-pigment for UV sensitivity of Opn5A and Opn5B (Fig. 4c, d)

As an attempt, the least-squares fitting for ERG data with nomograms experimentally determined for Gq-rhodopsin, xenopsin, and Opn5A (Figs. 4a, b, and 5b), as well as that roughly estimated based on the wavelength of maximum responses for Opn5B (Fig. 4d), was performed. The ERG sensitivities could be successfully explained if the relative contributions of the four opsins (i.e. coefficients for Gq-rhodopsin, xenopsin, Opn5A, and Opn5B nomograms in the least squares fitting, see Methods) were supposed to be 1.5, − 0.69, 1.3 and − 0.62 for the dark-adapted state and 2.0, − 1.2, 0.47 and − 0.38 for the light-adapted state, respectively (Figs. 7, Additional file 10: Fig. S9). The coefficients could represent contributions of each opsin to the signal transduction and/or the light-induced membrane depolarization of photoreceptors.

Light adaptation is caused by various molecular mechanisms in the photoreceptors, including the translocation of β-arrestin, which reduces the available receptor amounts in the membranes of rhabdomere [63]. If there is a difference in the affinity to β-arrestin among these opsins, the change in the contribution of each opsin to ERG in the light-adapted state may be achieved.

Movement of filtering screening pigments is another possible mechanism underlying the shift in spectral sensitivity between dark and light adaptation, and has been reported in the eyes of some arthropods [64–66]. However, no visible colored pigments exist in the ocular components that intervene between the outside environment and rhabdomere, i.e., the cornea, lens, or vitreous body, although the pigment layer consisting of the melanin-containing pigment granules is present between the cell body and rhabdomeric layers (Fig. 2b). Therefore, unlike some arthropods’ eyes, the shift of the spectral sensitivity is irrelevant to filtering pigments in the eye of *Limax*.

Accordingly, the finding that spectral sensitivity of a single photoreceptor cell can be generated by multiple opsins with different spectral sensitivity and G protein activation selectivity may explain the fine-tuning of vision, like the spectral sensitivity between dark- and light-adapted states observed in *Limax*. Such a mechanism would provide a simple solution for fine-tuning of vision without the requirement of additional layers of neural networks. Further investigation into the behavior of opsins and phototransduction molecules may uncover the relationship between the spectral shift that depends on the light adaptation state and the presence of several kinds of opsins in a single photoreceptor cell in the terrestrial slug *Limax*.

Recently, we proposed that animal opsins with different molecular properties are available for optogenetic control of GPCR signaling [67]. The complex regulation of phototransduction involving multiple opsins in the slug photoreceptors might provide a clue to a fine optogenetic control method with multiple opsins for cellular signaling involving multiple GPCRs.

## Conclusions

We demonstrated the co-expression of four opsins coupling to Gq and/or Go signaling cascades in an eye photoreceptor of *Limax*. Presence of multiple opsins with distinct spectral sensitivities and G protein coupling partners may underlie the shift in the spectral sensitivity of ERG depending on the light-adaptation states.

## Methods

### Animals

Terrestrial slugs *Limax valentianus* were maintained in an incubator (19 °C) in our laboratory for at least 36 generations, as a closed colony. They were fed a diet of humidified powder mixture, consisting of 520 g of rat chow (Oriental Yeast, Tokyo, Japan), 500 g of potato starch, and 21 g of vitamin mixture (AIN-76, Oriental Yeast).

### Generation of anti-Opn5B antibody

A rabbit was immunized with C-terminal 14 amino acids of *Limax* Opn5B (PLKGVGKYSAENKD) conjugated to Keyhole limpet hemocyanin by Cys residue added to the N-terminus. The antibody was obtained from the antiserum by affinity purification using the peptide CPLKGVGKYSAENKD covalently attached to *N*-hydroxysuccinimide (NHS)-activated Sepharose 4 Fast Flow (GE Healthcare, Little Chalfont, UK). The concentration of the antibody was determined using a Pierce BCA protein assay kit (Thermo Scientific, Waltham, MA) according to the manufacturer’s instructions.

### Immunocytochemistry

To evaluate the reactivity of the antibody, cultured cells were immunocytochemically stained. HEK293 cells were cultured on a poly-L-lysine coated glass-bottom dish (MatTek, Ashland, MA) in Dulbecco’s modified Eagle medium (Wako, Osaka, Japan) supplemented with 10% fetal bovine serum at 37 °C in 5% CO_2_-95% air. The cells were transfected with the expression vector pcDNA4-HisMax (Thermo Scientific) harboring the ORFs of Opn5B or limGα_q_, using Lipofectamine 3000 (Thermo Scientific) according to the manufacturer’s instructions. In some experiments, the cells were transfected with pEGFP-c2 (Takara, Ohtsu, Japan) harboring the full ORF of limGα_o_. After 24 h, the cells were washed with ice-cold PBS, and fixed in 4% paraformaldehyde (in PBS) for 30 min, followed by permeabilization with PBS supplemented with 0.1% Triton X-100 (PBST) for 10 min. Following a brief wash in PBS, the cells were blocked in blocking buffer (PBST supplemented with 2.5% bovine serum albumin, 2.5% goat serum) for 3–7 h. The cells were then incubated with a primary antibody against Opn5B (0.1 μg/ml, see above), Gα_o_ (1:500 in blocking buffer, #551, MBL, Tokyo, Japan), or Gα_q_ (1:200 anti-Gα_11_ antibody in blocking buffer, ZRB1446, Sigma, St. Louis, MO) at 4 °C overnight. According to the manufacturers, the anti-Gα_o_ antibody was raised against bovine Gα_o_, which is 82% identical at the amino acid levels to limGα_o_, and anti-Gα_q_ antibody was raised against C-terminal 10 amino acids of human Gα_11_, which are 100% identical to those of limGα_q_. For the cells transfected with pcDNA-HisMax-Opn5B and pcDNA-HisMax-limGα_q_, the mouse monoclonal antibody against 6 × His tag (1:200, Proteintech, Rosemont, IL) was also added. After washing three times in PBS, the cells were incubated with Alexa488- or Alexa594-labeled anti-rabbit IgG antibody (1:500 in blocking buffer, Thermo Scientific) in blocking buffer for 1 h at room temperature. For the cells transfected with pcDNA-HisMax-Opn5B and pcDNA-HisMax-limGα_q_, Alexa594-labeled anti-mouse IgG antibody (1:500, Thermo Scientific) was supplemented. The cells were then washed in PBS, followed by incubation in 0.1 μg/ml DAPI in PBS for 15 min, and washed again in PBS. The fluorescence images were obtained using a confocal laser scanning microscope C2 (Nikon, Tokyo, Japan) equipped with a 40 × objective lens (NA 0.95).

### Western blotting

The HEK293 cells were cultured in 6-well culturing plates (Corning, Corning, NY) and transfected with the plasmid pEGFP-limGα_o_ as described above. Twenty-four hours after transfection, the cells were washed in ice-cold PBS and lysed in TNE (10 mM Tris (pH 7.5), 150 mM NaCl, 1 mM EDTA, 1% Nonidet P-40) supplemented with protease inhibitor cocktail (Wako). The concentrations of the protein were determined using a Pierce BCA protein assay kit. The cell lysates were mixed with an equal volume of 2 × SDS sample buffer (50 mM Tris (pH 6.8), 4% SDS, 10% glycerol, 10% β-mercaptoethanol, 0.01% bromophenol blue) and boiled at 100 °C for 3 min. The samples (5 μg protein) were electrophoresed in the SDS–polyacrylamide gel (running gel of 10% polyacrylamide). The separated protein was electrically transferred to a nitrocellulose membrane and blocked in 5% skim milk dissolved in T-TBS (20 mM Tris (pH 7.5), 137 mM NaCl, 0.2% Tween-20) for 4–5 h at room temperature. The membrane was incubated with the primary antibody (1:2000 anti-Gα_o_ antibody (MBL) or 1:10,000 anti-α-tubulin mouse monoclonal antibody (Sigma)) dissolved in T-TBS at 4 °C overnight. After washing three times in T-TBS, the membrane was incubated with horseradish peroxidase-conjugated secondary antibody (1:10,000) against rabbit IgG (GE Healthcare) or mouse IgG (GE Healthcare) for 1 h at room temperature. After washing three times in T-TBS, the signals were visualized using Immunostar LD (Wako) and LuminoGraph-I (Atto, Tokyo, Japan).

### Immunohistochemistry

The slug was deeply anesthetized by an injection of Mg^2+^ buffer and chilled on ice for 1 min to ensure complete anesthetization. For immunostaining of xenopsin, Opn5A, and Opn5B, the left ST was isolated and frozen in Tissue-Tek Optimal cutting temperature compound (Sakura Finetek, Tokyo, Japan) using liquid nitrogen. The sections (10 μm thick) were cut using a cryostat, mounted onto glass slides (CREST micro slide glass, Matsunami, Osaka, Japan), and fixed in 4% paraformaldehyde (in PBS) supplemented with 0.1% glutaraldehyde for 30 min. In the experiment of Fig. 2, the sections (6 μm thick) were fixed in 4% paraformaldehyde (in PBS) for 30 min. For immunostaining of Gα_q_, the isolated left ST was fixed in 4% paraformaldehyde (in PBS) for 1 h, followed by a wash in PBS. The fixed ST was frozen in Tissue-Tek Optimal cutting temperature compound using liquid nitrogen. The sections (14 μm thick) were cut and mounted onto CREST micro slide glasses and post-fixed for 20 min in neutralized formalin (Nakalai-Tesque, Kyoto, Japan) diluted to 5% with PBS. For immunostaining of Gα_o_, the isolated ST was fixed in 4% *N*-(3-dimethylaminopropyl)-*N*’-ethylcarbodiimide hydrochloride (EDAC)/0.4% NHS dissolved in 0.1 M phosphate buffer (pH 7.4) for 1 h, followed by a wash in PBS. The fixed tissue was frozen in Tissue-Tek Optimal cutting temperature compound using liquid nitrogen. The sections were cut (14 μm thick), mounted onto CREST micro slide glasses, and post-fixed in neutralized formalin diluted to 5% with PBS for 20 min. The sections were permeabilized in PBST for 10 min, followed by a block in blocking buffer for 3–8 h. The sections were then incubated with the primary antibody in blocking buffer at 4 °C overnight. The concentrations of the primary antibodies were 0.2, 0.3, 1.0, and 0.1 μg/ml for Gq-rhodopsin, xenopsin, Opn5A, and Opn5B, respectively. The specificities of antibodies against Gq-rhodopsin, xenopsin, and Opn5A have already been characterized [6]. The dilution rates of the primary antibodies for G proteins were 1:200 for Gα_q_ (ZRB1446, Sigma), and 1:500 for Gα_o_ (#551, MBL). To further confirm the specificity of the primary antibody against limGα_o_, the antibody was pre-adsorbed with 0.1 μg/μl recombinantly expressed limGα_o_ fused to the C-terminus of MPB (MBP-limGα_o_), which was bacterially expressed from the pMAL-c2 vector (NEB, Ipswich, MA) and purified using amylose resin (NEB) according to the manufacturer’s instruction. Pre-adsorption was performed at 4 °C overnight. Following incubation with primary antibodies, the sections were washed three times in PBS and incubated with secondary anti-rabbit IgG (1:500, Alexa488-labeled) in blocking buffer for 1 h at room temperature. In some experiments, Alexa594-labeled streptavidin (1:1000, Thermo Scientific) was added to the secondary antibody to visualize the apical protrusion of Type-I eye photoreceptors [6]. After a wash in PBS, the sections were incubated with 0.1 μg/ml DAPI in PBS for 15 min, followed by a wash in PBS. The sections were coverslipped with Fluoromount (SouthernBiotech, Birmingham, AL). The fluorescence images were acquired using an Eclipse E600 microscope (Nikon) equipped with a DP70 CCD camera (Olympus, Tokyo, Japan) and a 20 × (NA 0.50) or 40 × (NA 0.75) objective lens.

### Electron microscopy

Electron microscopy was performed as described previously with slight modifications [63]. Briefly, an isolated left ST was fixed in 3% paraformaldehyde/1% glutaraldehyde dissolved in 20 mM phosphate buffer (pH 7.3) for 2 h at room temperature. The fixed ST was rinsed with 0.1 M phosphate buffer (pH 7.4), followed by osmification and dehydration, and embedded in epoxy resin. Ultra-thin sections were cut and stained with uranyl acetate and lead citrate. Images were obtained using a transmission electron microscope, JEM-1400plus (JEOL, Tokyo, Japan).

### In situ hybridization

The frozen sections (14 μm thick) of the left ST and brain were prepared as described above. The fixation, hybridization, and washing were performed as described previously [68]. Digoxigenin-labeled cRNA probes were generated by in vitro transcription as described previously [69]. The regions of the cRNA probes corresponded to the 608–1134 bases of limGα_o_ and 1137–1610 bases of limGα_i_. The concentrations of antisense and sense probes were adjusted so that both probes had equivalent titers. Fluorescence signals were detected using SigmaFast FastRed TR Naphthol AS-MX tablets (Sigma) as described previously [70], and the same reaction times were given for antisense and sense probes. The images were obtained using an Eclipse 600 fluorescence microscope equipped with a DP70 CCD camera and a 10 × (NA 0.45) or 20 × (NA 0.50) objective lens. The images of the fluorescence signals with antisense and sense probes were acquired at the same exposure times.

### GloSensor assay and heterologous action spectroscopy

To estimate spectral sensitivities of the opsin-based pigments, light-induced changes in [cAMP]_i_ of the opsin-expressing HEK293S cells were measured by GloSensor cAMP assay (Promega, Fitchburg, WI) as described previously [30, 57]. Briefly, the expression vector pcDNA3.1 (Thermo Scientific) harboring cDNA of Gq-rhodopsin or xenopsin chimeras, whose third intracellular domain was replaced with Gs-coupled jellyfish opsin [32] or the full-length cDNA of Opn5A, Opn5B or retinochrome, were introduced into HEK293S cells, together with the pGloSensor-22F plasmid (Promega), using the polyethylenimine transfection method. All opsin constructs were C-terminally tagged with the monoclonal antibody rho 1D4 epitope sequence (ETSQVAPA). The luminescence, indicating a change in [cAMP]_i_, was monitored using a GloMax 20/20n luminometer (Promega). For Gq-rhodopsin and xenopsin, spectral sensitivity curves were examined by HAS [30, 31]. Briefly, relative sensitivities at 410, 430, 470, 510, and 540 nm were obtained using LEDs as light sources (5.8 × 10^13^ photon cm^2^/sec), and the sensitivity values were fitted with a rhodopsin template, rhodopsin nomogram [34]. For Opn5A and Opn5B, relative responses were obtained based on decreases of [cAMP]_i_ induced by irradiation with equal photons of 370, 410, 430, 470, 510, 540, and 580 nm light from LEDs, in Opn5A or Opn5B-expressing HEK293 cells as reported previously [33]. We also investigated the signaling capacity of retinochrome by monitoring the change in [cAMP]_i_ and [Ca^2+^]_i_ in response to green light. Bovine rhodopsin and jumping spider rhodopsin [71] were used as positive controls, respectively. The change in [Ca^2+^]_i_ was monitored by aequorin-based luminescence assay as described previously [71].

### Expression and purification of the opsin-based pigment and spectroscopy

The opsin expression and purification were performed essentially as described previously [72]. Briefly, expression vectors pMT obtained from Addgene (Addgene plasmid 15,896), harboring xenopsin or Opn5A, were transfected into HEK293S cells using the calcium-phosphate method. After the addition of the 11-*cis* retinal, opsin-based pigments were extracted with 1% dodecyl β-D-maltoside and purified with the rho 1D4 antibody. The absorption spectra of the pigment were recorded at 4 °C using a V-750 UV–VIS spectrophotometer (JASCO International, Japan). Blue and orange light were irradiated using a halogen light with a 480-nm interference filter and an O58 glass cutoff filter (Toshiba, Tokyo, Japan), respectively.

### NanoBiT assay

Light-dependent G protein activations by opsins were investigated by NanoBiT-G-protein dissociation assay [36]. Briefly, the expression vector pcDNA3.1 (Thermo Scientific) containing opsins was introduced into HEK293S cells, together with human Gα_o1_-LgBit or Gα_q_-LgBit, Gβ1-SmBit and Gγ2 expression vectors, using the polyethylenimine transfection method. The decrease in luminescence, indicating dissociation of Gα from Gβγ, was analyzed using a GloMax 20/20n luminometer. A broadband green LED light was applied for 5 s as a light stimulus.

### Fitting of ERG data by rhodopsin nomograms

Rhodopsin nomograms for Gq-rhodopsin and xenopsin were determined based on their absorption spectra obtained by HAS. The rhodopsin nomogram for Opn5A in the visible region was determined based on the absorption spectrum and that in the UV region was estimated based on the UV response of Opn5A-expressing cultured cells. The rhodopsin nomogram for Opn5B was estimated based on the wavelength of maximum light response in the visible region and the UV response of Opn5B-expressing cells. Note that 360 nm-pigment nomograms of Opn5A and Opn5B were integrated into the calculation with the same coefficients as those for the respective visible sensitivities. The unconstrained summation of four rhodopsin nomograms was applied to the least-squares fitting for the ERG data. The function with wavelength (λ) as an independent variable is as follows: f(λ) = A*e^(-380*log (λ/510)^2*(1 + 6.09*log (λ/510) + 13.9*log (λ/510)^2)) + B*e^(-380*log (λ/470)^2*(1 + 6.09*log (λ/470) + 13.9*log (λ/470)^2)) + (A + B)*e^(247*log (λ/360)^2*(1 + 3.59*log (λ/360) + 4.83*log (λ/360)^2)) + Rh*e^(-380*log (λ/456)^2*(1 + 6.09*log (λ/456) + 13.9*log (λ/456)^2)) + Xe*e^(-380*log (λ/474)^2*(1 + 6.09*log (λ/474) + 13.9*log (λ/474)^2)), where A, B, Rh and Xe are coefficients for nomograms of Opn5A, Opn5B, Gq-rhodopsin and xenopsin, respectively.

### Supplementary Information

**Additional file 1:** **Fig. S1.** The validity of the anti-Opn5B antibody was confirmed by dual fluorescence immunocytochemistry of the HEK293 cells transfected with pcDNA-HisMax-Opn5B. Fluorescence signals of Opn5B immunoreactivity were completely overlapped with those of 6×His immunoreactivity. (**a**) Immunohistochemical staining with anti-Opn5B antibody. (**b**) Immunoreactive signals with anti-6×His monoclonal antibody. (**c) **A merged image of (a) and (b), superimposed on the fluorescence signals of DAPI (blue). Scale bar: 50 μm.

**Additional file 2:** **Fig. S2. **Applicability of commercially obtained anti-Gα protein antibodies (anti-human Gα_11_ and anti-bovine Gα_o_ antibodies) was confirmed using HEK293 cells transfected with pcDNA-HisMax-limGα_q_ or pEGFP-c2-limGα_o_. (**a**-**c**) Anti-human Gα_11_ antibody recognized the HEK293 cells transfected with pcDNA-HisMax-limGα_q_, which expresses Gα_q_ of *Limax* (limGα_q_) N-terminally tagged with 6×His, and its signals overlapped with those detected using anti-6×-His antibody. Nuclear signal of DAPI is shown in (**c**). (**d**-**f**) HEK293 cells expressing Gα_o_ of *Limax* (limGα_o_) N-terminally tagged with an enhanced green fluorescent protein (GFP) were visualized with (d) anti-bovine Gα_o_ antibody and (e) GFP fluorescence. Both signals overlapped. Nuclear signal of DAPI is shown in (f). (**g**) Western blotting of the cell lysates further confirmed that the antibody exhibits a single band in the lysate of the HEK293 cells transfected with pEGFP-c2-limGα_o_ with a molecular mass that was roughly consistent with the predicted value (approx. 70 kDa). Five μg of protein were loaded on each lane. Immunoblotting with anti-α-tubulin antibody is displayed below as a loading control. Scale bar: 50 μm.

**Additional file 3:** **Fig. S3. **The mRNA of the alpha subunit of Gi (limGα_i_) was not detected in the retina. (**a**-**e**) Expression of limGα_i_ in the brain of *Limax*. Antisense (a), but not the sense probe (c), exhibited the signal of limGα_i_ in the pleural and parietal ganglia in the brain. (b) and (d) are the fluorescence images of DAPI of (a) and (c). White arrow heads indicate the signals in the left pleural ganglion and right parietal ganglion. (**e**) A cartoon of the dorsal view of the brain, indicating the areas of the micrographs (circumscribed with a red broken line). (**f**-**j**) No signal was detected with either antisense (f) or sense (h) probes in the superior tentacle. (g) and (i) are the fluorescence images of DAPI for (f) and (h).** (j**) A cartoon of the lateral view of the superior tentacle corresponding to the micrographs. Note that images of *in situ *hybridization signals (a, c, f, h) were all acquired using the same exposure time. Scale bar: 200 μm. A, anterior; P, posterior; R, right; L, left; D, dorsal; V, ventral. PC, procerebrum; CG, cerebral ganglion; PlG, pleural ganglion; PaG, parietal ganglion; PeG, pedal ganglion; ViG, visceral ganglion; TN, tentacular nerve; ON, optic nerve; AR, accessory retina; TG, tentacular ganglion; PL, pigment layer.

**Additional file 4:** **Fig. S4.** The dose-response (light intensity-response) for cultured cells expressing opsins (**a**) *Limax* Gq-coupled rhodopsin. (**b**) *Limax* xenopsin. The intensity-response curves were obtained by fitting a sigmoid function to relative responses to the 500 nm light stimulus at five intensities spanning a 1000-fold range in intensity.

**Additional file 5:** **Table S1.** Individual peak values of relative fluorescence in GloSensor assay (each n=3).

**Additional file 6:** **Fig. S5. **Examples of responses of mock-transfected HEK293S to UV and blue light. Light-dependent changes in cAMP level were not detected by GloSensor assay for either light (n=3, ±SE).

**Additional file 7:** **Fig. S6. **Inability of retinochrome to activate phototranduction. (**a**) Bovine rhodopsin but not *Limax* retinochrome induced a decrease in [cAMP]i by green light. (**b**) Jumping spider Rh1 but not *Limax* retinochrome induced a rise in [Ca^2+^]i by green light. Note that retinochromes generally form visible light sensitive pigments [19, 73–75], which absorb light supplied by the broadband green LED (Additional file 11: Fig. S10b).

**Additional file 8:** **Fig. S7.** Individual data in the NanoBit-G protein dissociation assay (n=3) of Gq/Go activation by opsins.

**Additional file 9:** **Fig. S8. **A negative control experiment for NanoBiT assay of Go. In the NanoBiT assay of Gs (left), a light-dependent decrease of luminescence was observed in the presence of Jellyfish opsin (red solid line) but not in its absence (mock, black solid line), indicating jellyfish opsin activates Gs, which is consistent with the previous report [32]. On the other hand, in the NanoBiT assay of Go (right), there was almost no difference in the rate (slope) of luminescence decrease between before and after light irradiation in the presence of jellyfish opsin (red solid line), showing that jellyfish opsin does not activate Go in a light-dependent manner. Note that the light-“independent” decrease of luminescence could be due to temperature or other factors, but it was not clear. When the slope is corrected so that the light-independent decrease in luminescence before irradiation is eliminated (red dotted line), the profiles before and after light irradiation are almost identical to those in the absence of jellyfish opsin (mock, black solid line), supporting the above explanation that no light-dependent luminescence change occurs. These observations exclude the possibility that Go is promiscuously activated by any opsin in this NanoBiT assay system.

**Additional file 10:** **Fig. S9.** Rhodopsin nomograms used for fitting the ERG data. Rhodopsin nomograms for Gq-rhodopsin (black curve), xenopsin (green curve), Opn5A (red curve) and Opn5B (blue). See also Fig. 7 and Methods for details.

**Additional file 11:** **Fig. S10.** Spectra of LED used in GloSensor and NanoBit assays. (**a**) From left to right, spectra of 370 nm, 410 nm, 430 nm, 470 nm, 510 nm, 540 nm and 580 nm monochromatic lights used were shown. (**b**) The spectrum of broadband green LED light.

## Data Availability

The nucleotide sequences of limGα_q_, limGα_o_, and limGα_i_ were submitted to DDBJ under the accession numbers LC717977, LC717978, and LC717979, respectively. All data generated or analyzed during this study are included in this published article (Figs. 1, 2, 3, 4, 5, 6 and 7) and its supplementary information files (Additional files 1–11). Materials can be provided upon reasonable request.

## Abbreviations

CCD: Charge-coupled device
DAPI: 4′6-Diamidino-2-phenylidole
ERG: Electroretinogram
HAS: Heterologous action spectroscopy
HEPES: 2-[4-(2-Hydroxyethyl)-1-piperazineyl]ethanesulfonic acid
MBP: Maltose binding protein
ORF: Open reading frame
PBS: Phosphate-buffered saline
SDS: Sodium dodecyl sulfate
SE: Standard error
ST: Superior tentacle
