# Supplementary figures and images for: Functional characterization of four opsins and two G alpha subtypes co-expressed in the molluscan rhabdomeric photoreceptor
Source: BMC Biol. 2023 Dec 18;21:291. doi: 10.1186/s12915-023-01789-7 (PMC10729476; doi:10.1186/s12915-023-01789-7)

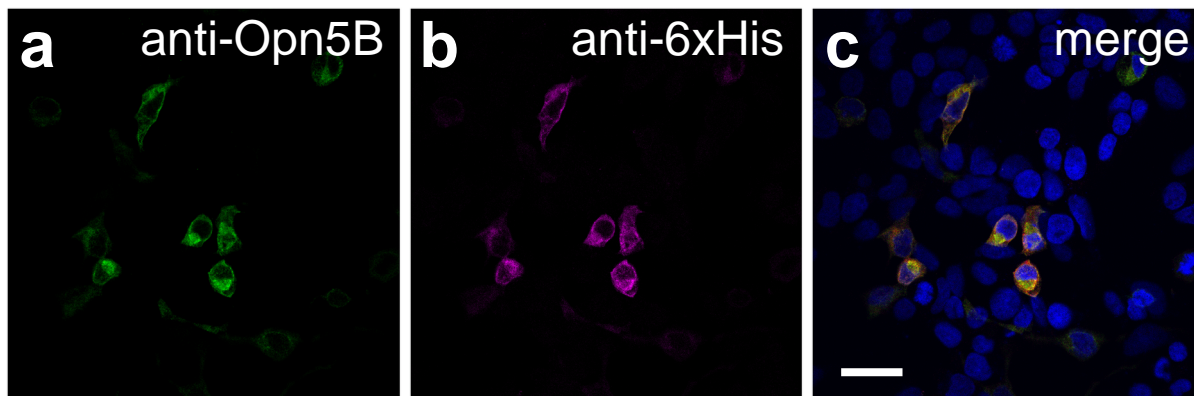

Figure S1

Supplement: Supplementary file 1 — Additional file 1: Fig. S1. The validity of the anti-Opn5B antibody was confirmed by dual fluorescence immunocytochemistry of the HEK293 cells transfected with pcDNA-HisMax-Opn5B. Fluorescence signals of Opn5B immunoreactivity were completely overlapped with those of 6×His immunoreactivity. (a) Immunohistochemical staining with anti-Opn5B antibody. (b) Immunoreactive signals with anti-6×His monoclonal antibody. (c) A merged image of (a) and (b), superimposed on the fluorescence signals of DAPI (blue). Scale bar: 50 μm. [file 12915_2023_1789_MOESM1_ESM.pdf]

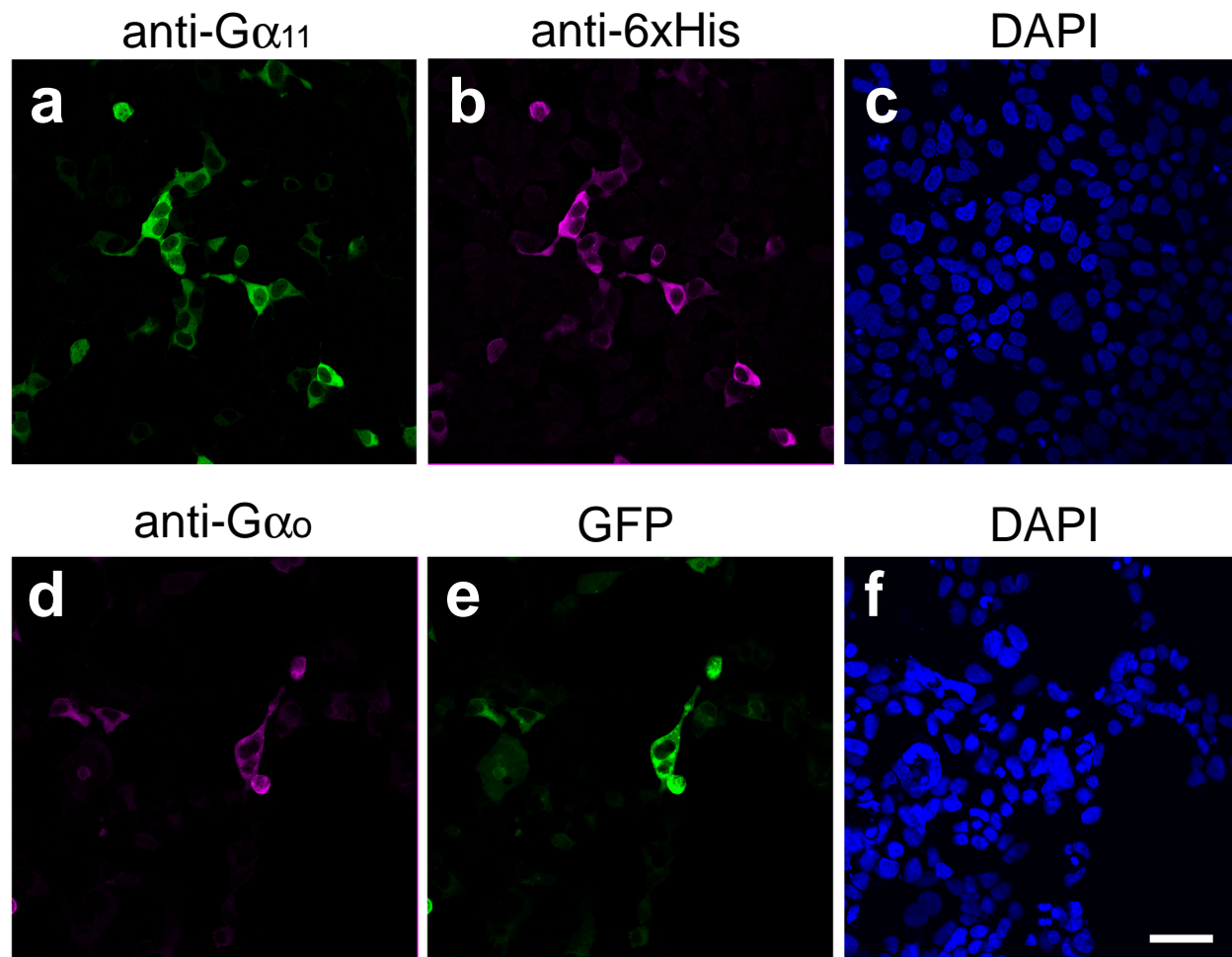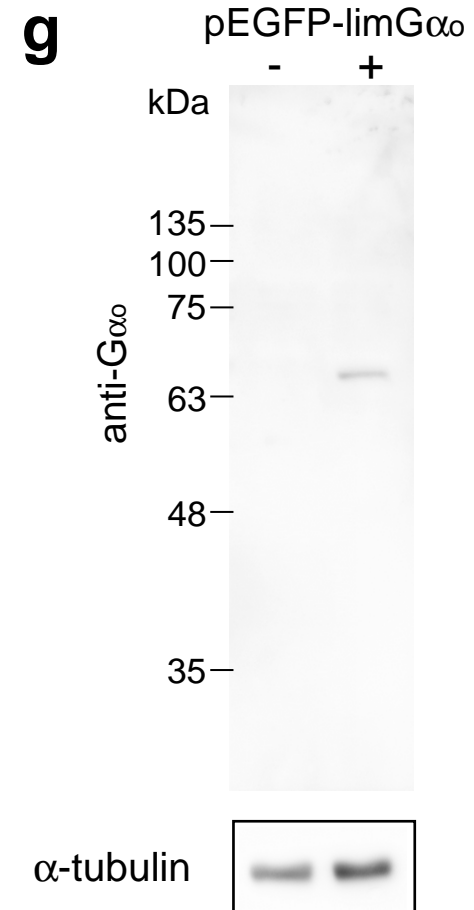

Figure S2

Supplement: Supplementary file 2 — Additional file 2: Fig. S2. Applicability of commercially obtained anti-Gα protein antibodies (anti-human Gα11 and anti-bovine Gαo antibodies) was confirmed using HEK293 cells transfected with pcDNA-HisMax-limGαq or pEGFP-c2-limGαo. (a-c) Anti-human Gα11 antibody recognized the HEK293 cells transfected with pcDNA-HisMax-limGαq, which expresses Gαq of Limax (limGαq) N-terminally tagged with 6×His, and its signals overlapped with those detected using anti-6×-His antibody. Nuclear signal of DAPI is shown in (c). (d-f) HEK293 cells expressing Gαo of Limax (limGαo) N-terminally tagged with an enhanced green fluorescent protein (GFP) were visualized with (d) anti-bovine Gαo antibody and (e) GFP fluorescence. Both signals overlapped. Nuclear signal of DAPI is shown in (f). (g) Western blotting of the cell lysates further confirmed that the antibody exhibits a single band in the lysate of the HEK293 cells transfected with pEGFP-c2-limGαo with a molecular mass that was roughly consistent with the predicted value (approx. 70 kDa). Five μg of protein were loaded on each lane. Immunoblotting with anti-α-tubulin antibody is displayed below as a loading control. Scale bar: 50 μm. [file 12915_2023_1789_MOESM2_ESM.pdf]

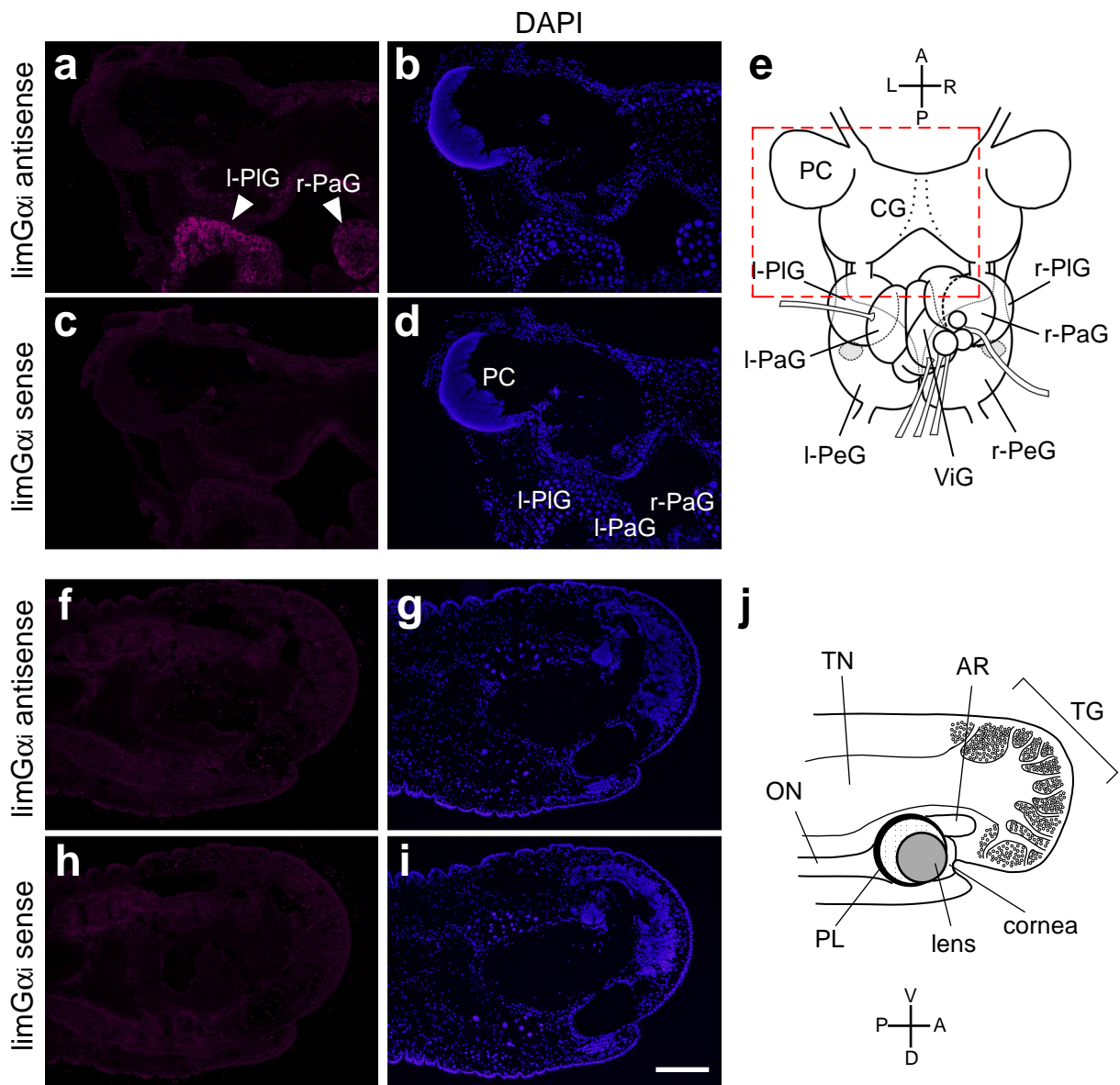

Figure S3

Supplement: Supplementary file 3 — Additional file 3: Fig. S3. The mRNA of the alpha subunit of Gi (limGαi) was not detected in the retina. (a-e) Expression of limGαi in the brain of Limax. Antisense (a), but not the sense probe (c), exhibited the signal of limGαi in the pleural and parietal ganglia in the brain. (b) and (d) are the fluorescence images of DAPI of (a) and (c). White arrow heads indicate the signals in the left pleural ganglion and right parietal ganglion. (e) A cartoon of the dorsal view of the brain, indicating the areas of the micrographs (circumscribed with a red broken line). (f-j) No signal was detected with either antisense (f) or sense (h) probes in the superior tentacle. (g) and (i) are the fluorescence images of DAPI for (f) and (h). (j) A cartoon of the lateral view of the superior tentacle corresponding to the micrographs. Note that images of in situ hybridization signals (a, c, f, h) were all acquired using the same exposure time. Scale bar: 200 μm. A, anterior; P, posterior; R, right; L, left; D, dorsal; V, ventral. PC, procerebrum; CG, cerebral ganglion; PlG, pleural ganglion; PaG, parietal ganglion; PeG, pedal ganglion; ViG, visceral ganglion; TN, tentacular nerve; ON, optic nerve; AR, accessory retina; TG, tentacular ganglion; PL, pigment layer. [file 12915_2023_1789_MOESM3_ESM.pdf]

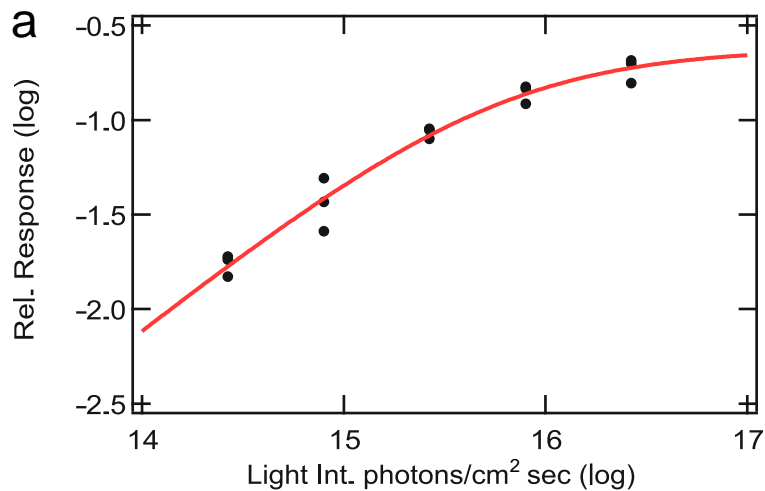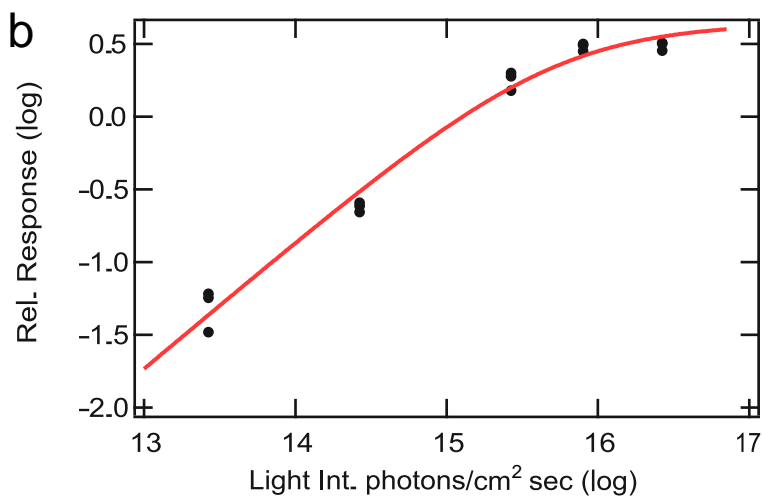

Figure S4

Supplement: Supplementary file 4 — Additional file 4: Fig. S4. The dose-response (light intensity-response) for cultured cells expressing opsins (a) Limax Gq-coupled rhodopsin. (b) Limax xenopsin. The intensity-response curves were obtained by fitting a sigmoid function to relative responses to the 500 nm light stimulus at five intensities spanning a 1000-fold range in intensity. [file 12915_2023_1789_MOESM4_ESM.pdf]

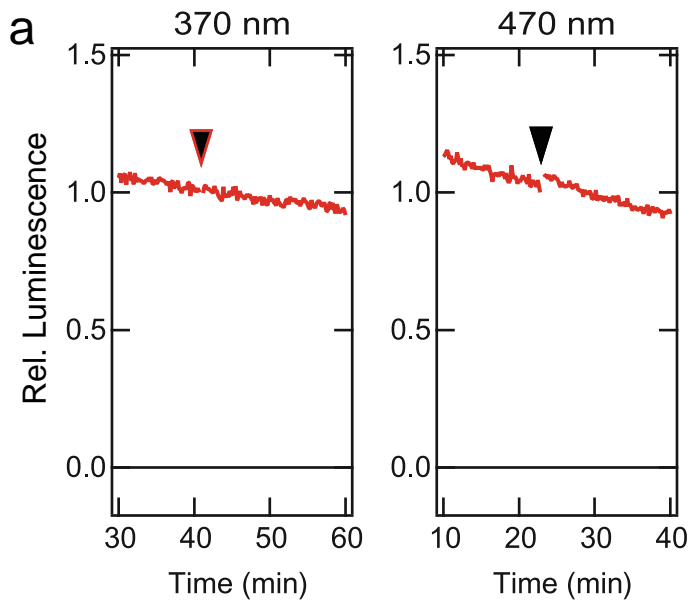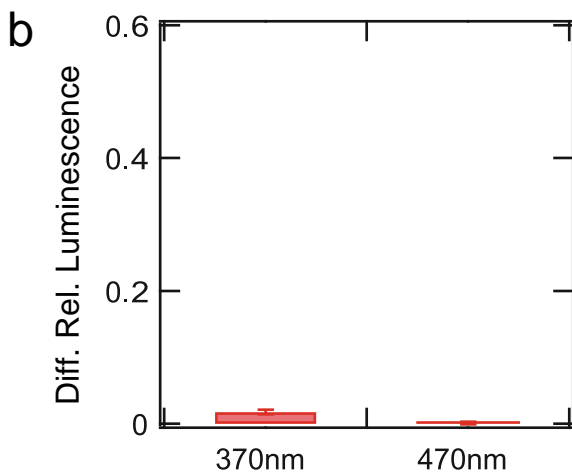

Figure S5

Supplement: Supplementary file 6 — Additional file 6: Fig. S5. Examples of responses of mock-transfected HEK293S to UV and blue light. Light-dependent changes in cAMP level were not detected by GloSensor assay for either light (n=3, ±SE). [file 12915_2023_1789_MOESM6_ESM.pdf]

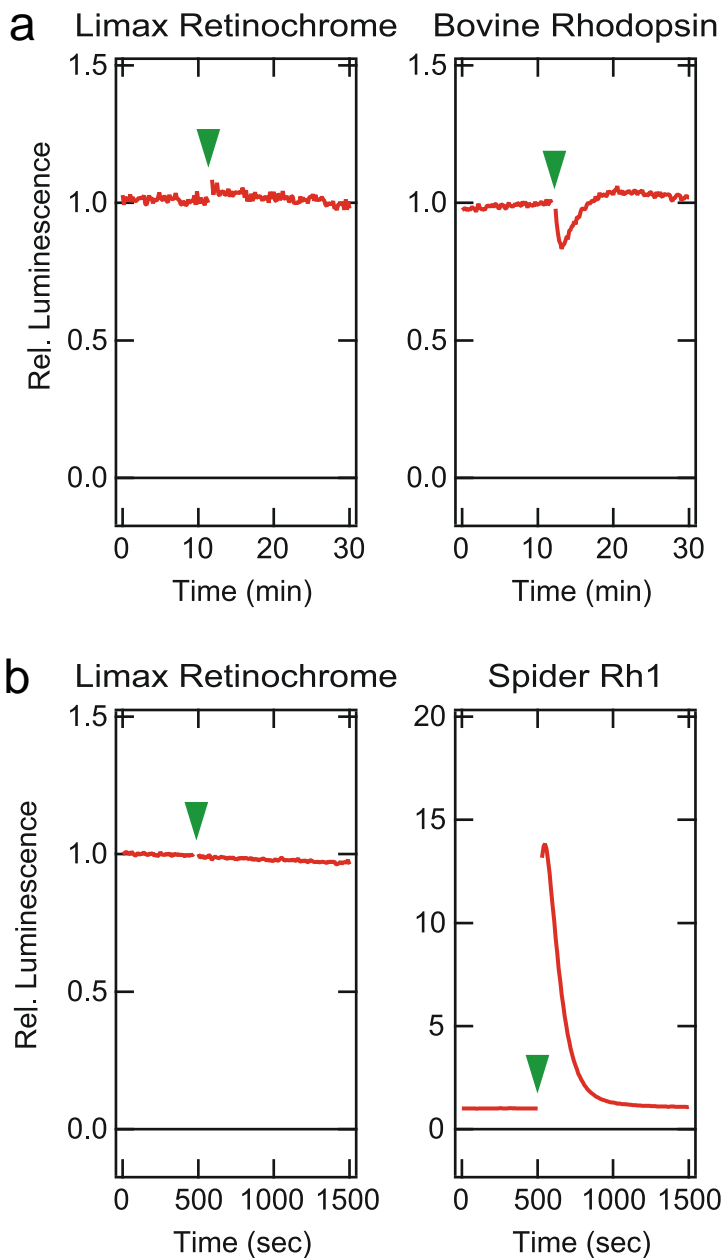

Figure S6

Supplement: Supplementary file 7 — Additional file 7: Fig. S6. Inability of retinochrome to activate phototranduction. (a) Bovine rhodopsin but not Limax retinochrome induced a decrease in [cAMP]i by green light. (b) Jumping spider Rh1 but not Limax retinochrome induced a rise in [Ca2+]i by green light. Note that retinochromes generally form visible light sensitive pigments [19, 73–75], which absorb light supplied by the broadband green LED (Additional file 11: Fig. S10b). [file 12915_2023_1789_MOESM7_ESM.pdf]

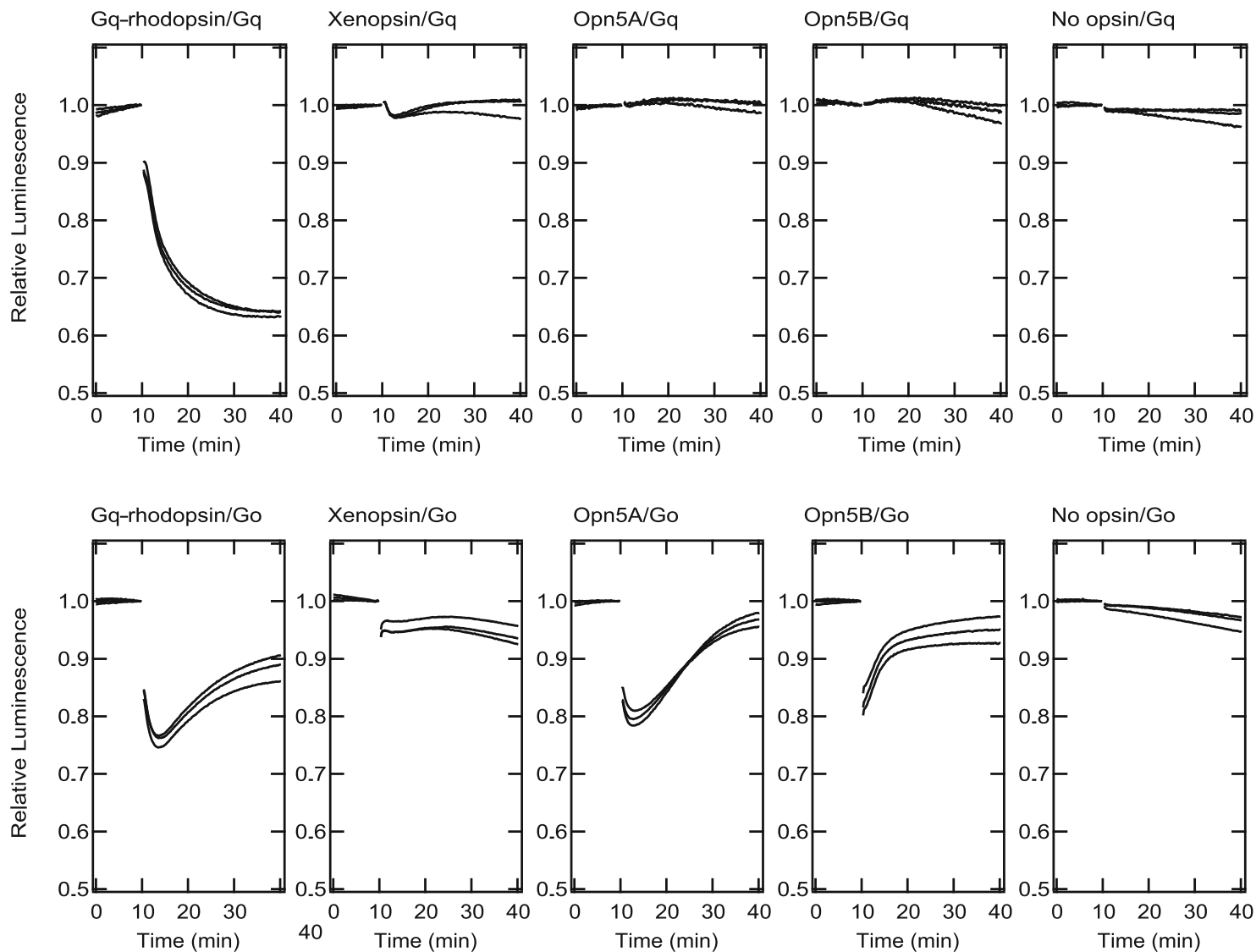

Fig. S7

Supplement: Supplementary file 8 — Additional file 8: Fig. S7. Individual data in the NanoBit-G protein dissociation assay (n=3) of Gq/Go activation by opsins. [file 12915_2023_1789_MOESM8_ESM.pdf]

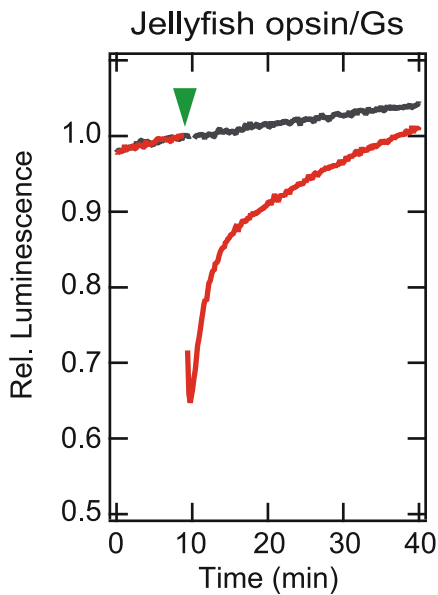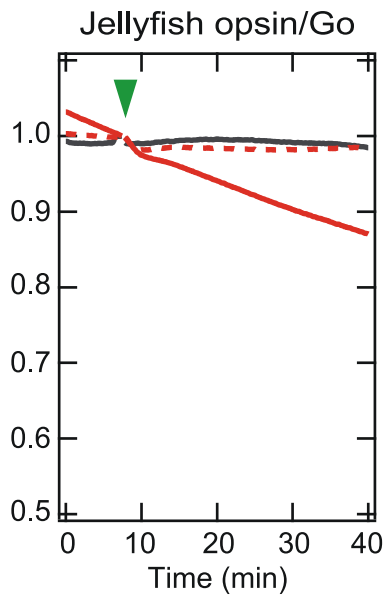

Figure S8

Supplement: Supplementary file 9 — Additional file 9: Fig. S8. A negative control experiment for NanoBiT assay of Go. In the NanoBiT assay of Gs (left), a light-dependent decrease of luminescence was observed in the presence of Jellyfish opsin (red solid line) but not in its absence (mock, black solid line), indicating jellyfish opsin activates Gs, which is consistent with the previous report [32]. On the other hand, in the NanoBiT assay of Go (right), there was almost no difference in the rate (slope) of luminescence decrease between before and after light irradiation in the presence of jellyfish opsin (red solid line), showing that jellyfish opsin does not activate Go in a light-dependent manner. Note that the light-“independent” decrease of luminescence could be due to temperature or other factors, but it was not clear. When the slope is corrected so that the light-independent decrease in luminescence before irradiation is eliminated (red dotted line), the profiles before and after light irradiation are almost identical to those in the absence of jellyfish opsin (mock, black solid line), supporting the above explanation that no light-dependent luminescence change occurs. These observations exclude the possibility that Go is promiscuously activated by any opsin in this NanoBiT assay system. [file 12915_2023_1789_MOESM9_ESM.pdf]

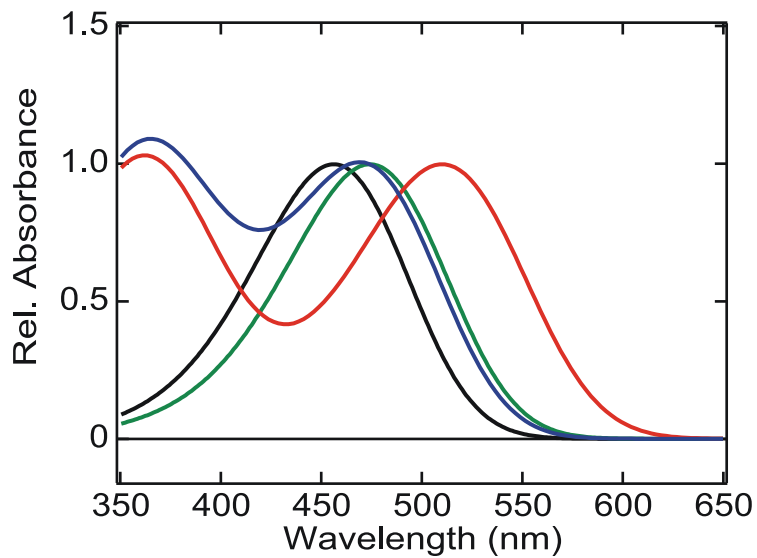

Figure S9

Supplement: Supplementary file 10 — Additional file 10: Fig. S9. Rhodopsin nomograms used for fitting the ERG data. Rhodopsin nomograms for Gq-rhodopsin (black curve), xenopsin (green curve), Opn5A (red curve) and Opn5B (blue). See also Fig. 7 and Methods for details. [file 12915_2023_1789_MOESM10_ESM.pdf]

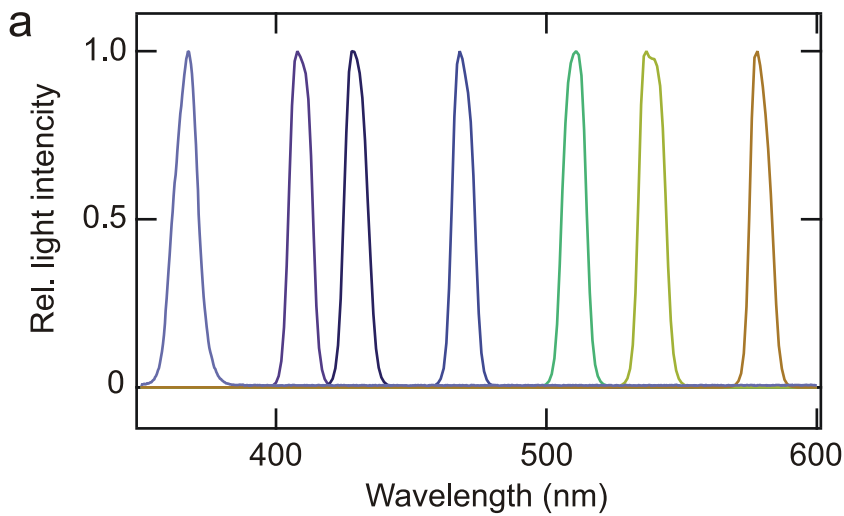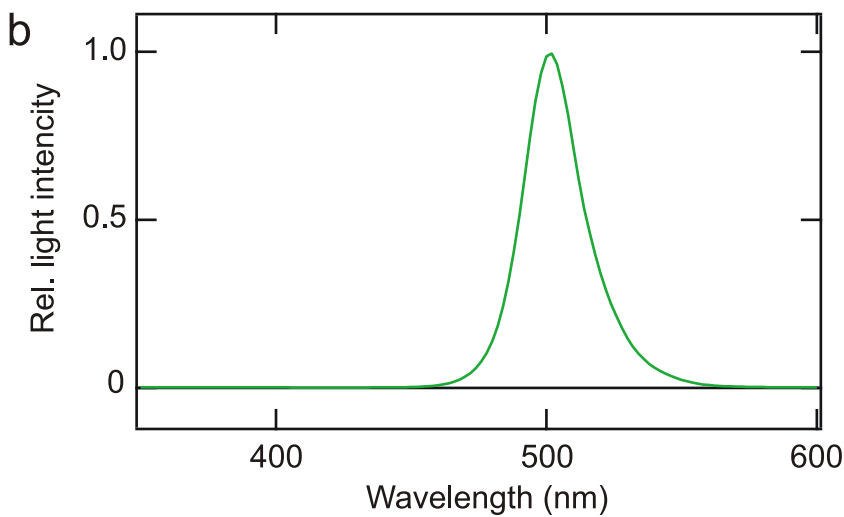

Figure S10

Supplement: Supplementary file 11 — Additional file 11: Fig. S10. Spectra of LED used in GloSensor and NanoBit assays. (a) From left to right, spectra of 370 nm, 410 nm, 430 nm, 470 nm, 510 nm, 540 nm and 580 nm monochromatic lights used were shown. (b) The spectrum of broadband green LED light. [file 12915_2023_1789_MOESM11_ESM.pdf]
